# Genetic Monitoring of Tibial Hemimelia in Galloway Cattle

**DOI:** 10.1002/age.70196

**Published:** 2026-09-02

**Authors:** Bertram Brenig

**Affiliations:** ^1^ Institute of Veterinary Medicine Georg‐August‐University Göttingen Göttingen Germany

Tibial hemimelia is a recessive developmental defect reported in various cattle breeds. The first reports of the defect date back to 1951 (Young 1951). Subsequent case reports appeared for Galloway cattle in 1974, 1977, 1978 and 1979 (Leipold et al. 1977, 1978; Ojo et al. 1974; Pollock et al. 1979). While the underlying genetic defect in Shorthorns was identified in 2012, the causative genetic variant in Galloway cattle was not elucidated until 2015 (Beever and Marron 2012; Brenig et al. 2015, 2024; Buitkamp et al. 2023). In Galloway cattle, tibial hemimelia is caused by a 20 bp duplication in exon 2 of the Aristaless‐like Homeobox 4 gene (*ALX4*) (Brenig et al. 2015). As this is a lethal factor in Galloway cattle, all breeding animals have since been screened for this genetic defect prior to breeding. Only animals that are not carriers (= THF) of the variant allele are approved for breeding. To verify the current genotype and allele frequencies in the German Galloway population, we tested a total of 1586 animals of Galloway (*n* = 906), Belted Galloway (*n* = 279) and White Galloway (*n* = 401) for the variant in the *ALX4* gene. Genotyping was performed using the same PCR assay and scoring criteria as in 2015, supporting comparability of genotype calls across years (Brenig et al. 2015). Cohort comparability with respect to herd/region composition and familial clustering was not evaluated and could influence temporal comparisons, particularly in subpopulations with small effective sizes. A total of 1342 DNA samples were successfully genotyped. For 244 animals, the genotype could not be determined due to poor DNA quality. Of the 1342 cattle, 1220 were THF and 122 were carriers of the variant (= THC). The genotypes of the individual breeds are shown in Figure 1. The average allele frequency of the variant allele within the 1342 DNA samples was 0.046. However, within the three Galloway breeds allele frequencies differed markedly, at 0.019 (Galloway = GAL), 0.009 (Belted Galloway = BGA), and 0.119 (White Galloway = WGA).

**FIGURE 1:**
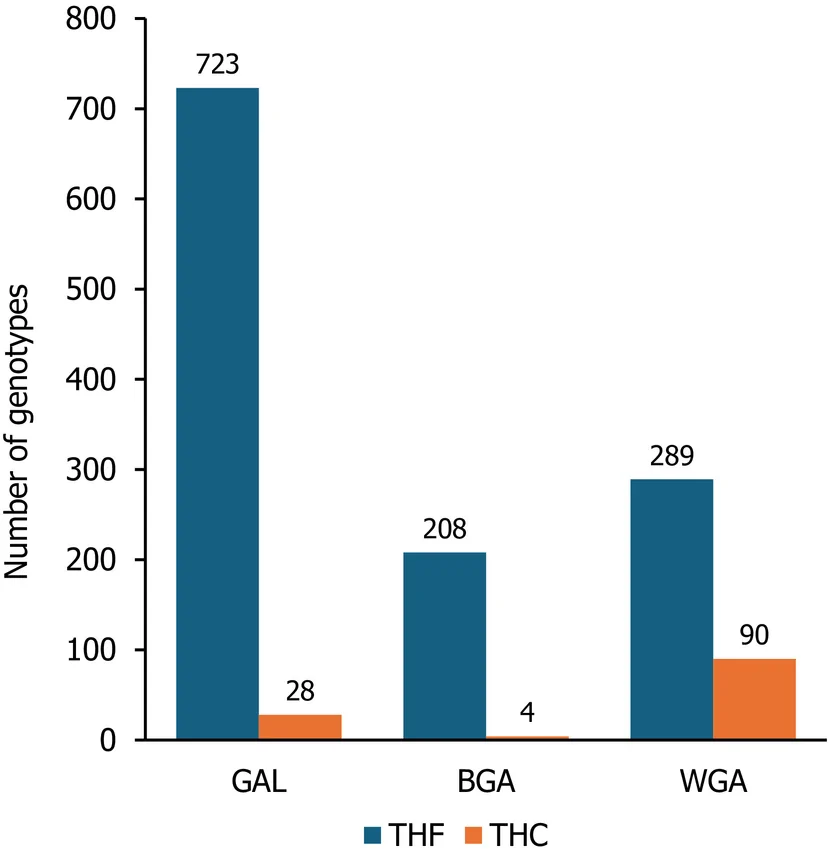
*ALX4* genotypes in Galloway cattle. A total of 1342 DNA samples of Galloway cattle (BGA = Belted Galloway, GAL = Galloway, WGA = White Galloway) were genotyped according to the described method (Brenig et al. 2015).

Compared with the earlier study (Brenig et al. 2015), the current allele frequency of the causative allele differs. The allele frequency for WGA in 2015 was 0.064, whereas in the current survey an allele frequency of 0.119 was determined. The 2015 study did not distinguish between the other Galloway breeds (GAL, BGA), but calculated an allele frequency of 0.014 for this group. If the data for GAL and BGA are combined in this study, the allele frequency is 0.017.

To examine changes in genotype frequencies from the time the genetic defect was first described (Brenig et al. 2015) to the present day, the number of genotypes from 2015 was compared with the current data in a 2 × 2 contingency table, and the *Χ* ^2^‐values were calculated (df = 1). A highly significant increase of heterozygotes in the WGA was observed in the current sample relative to 2015 (*p* < 0.001, *χ* ^2^ = 12.75); however, differences in cohort composition and familial clustering were not evaluated and could contribute. For Galloway and Belted Galloway combined, no significant change was observed (*p* = 0.44, *χ* ^2^ = 0.6). The significant increase in carriers in the WGA population is consistent with, but not directly demonstrated by, the breeding scheme in which selection for coat phenotype restricts effective population size; alternative explanations including popular‐sire founder effects and genetic drift cannot be ruled out from the current data (Brenig et al. 2013). There are three coat colour variants in White Galloways, known as well marked, strongly marked and mismarked. These variants result from duplication and aberrant insertion of the *KIT* gene from Chromosome 6 onto Chromosome 29 (Brenig et al. 2013; Durkin et al. 2012). The preferred coat colour variant is the well‐marked individual, which is heterozygous for the Chromosome 29 insertion. These well‐marked White Galloways can only be produced through selective mating of Black with mismarked Galloway. The availability of fully Black Galloways as breeding stock does not pose a problem; however, these matings limit the number of mismarked White Galloways available for other breeding. Due to selective breeding for the preferred coat colour phenotype (breeding bias) and associated reduction in the effective population size and increased inbreeding, the frequency of the recessive *ALX4* allele may increase over successive generations. Without selection against THC, there is no mechanism to remove it, and genetic drift in small populations can further increase its frequency.

Although a controlled reduction of the causative allele is theoretically achievable, its implementation in structured breeding systems where selection is based mainly on coat colour and no genetic testing on *ALX4* is performed does not achieve comparable effectiveness (reduction in allele frequency) or speed (reduction in allele frequency per generation) to random matings that include DNA‐based diagnosis. In the absence of molecular screening, the allele frequency remains stable or may even increase over time due to undetected propagation of heterozygous carriers, elevated inbreeding and the selective breeding of phenotypically preferred individuals that harbour the causative allele. In addition, although identifying carrier animals is necessary for animal welfare reasons, the data presented here are consistent with incomplete determination of the causative variant in breeding programmes, although a direct audit of individual screening records would be required to quantify programme non‐compliance and to distinguish it from alternative explanations such as time lag, imports or permitted carrier retention. This may be due to the fact that the variant cannot be reliably diagnosed using the bovine BeadChip and that the more expensive direct detection by PCR is still the only option. Although the overall allele frequency did not increase dramatically over the last 10 years, especially for Galloway and Belted Galloway, it remains advisable to more consistently identify carrier animals and exclude them from breeding.

## Author Contributions

**Bertram Brenig:** conceptualization, investigation, funding acquisition, writing – original draft, methodology, validation, visualization, writing – review and editing, formal analysis, project administration, data curation, resources.

## Conflicts of Interest

The author declares no conflicts of interest.

## Data Availability

Data sharing is not applicable to this article as no datasets were generated or analysed during this study.
